# The Current Status of Quality of Reporting in Acupuncture Treatment Case Reports: An Analysis of the Core Journal in Korea

**DOI:** 10.1155/2017/5810372

**Published:** 2017-05-31

**Authors:** Jeongjoo Kim, Yoon-Ji Eom, Ye-Seul Lee, Dongwoo Nam, Younbyoung Chae

**Affiliations:** ^1^Acupuncture and Meridian Science Research Center, College of Korean Medicine, Kyung Hee University, Seoul, Republic of Korea; ^2^Department of Acupuncture and Moxibustion, College of Korean Medicine, Kyung Hee University, Seoul, Republic of Korea

## Abstract

**Objectives:**

The present study aimed to evaluate the overall quality of case reports concerning acupuncture treatment in Korea.

**Methods:**

We selected a representative Korean journal and retrieved eligible case reports on acupuncture treatment published from 2009 to 2015. We assessed the quality of reporting based on CAse REport (CARE) and STandards for Reporting Interventions in Clinical Trials of Acupuncture (STRICTA) guideline checklists.

**Results:**

A total of 93 eligible case reports of acupuncture treatment were identified among the 107 articles screened. Overall quality of reporting in the case reports was generally acceptable (75.4% on CARE, 67.7% on STRICTA), but several crucial items remained substantially underreported.

**Conclusions:**

Endorsement of the CARE and STRICTA guidelines is needed to improve the completeness of reporting. Our findings will be helpful in developing a more appropriate reporting guideline for case reports in acupuncture treatment.

## 1. Introduction

Case reports are detailed narratives that describe a medical problem experienced by one or more patients for the purpose of medicine, science, or education [1]. They are considered useful for recognizing new diseases and identifying adverse events and beneficial effects associated with new treatments [2]. Since case reports are not sufficiently rigorous to show evidence of effectiveness in the era of clinical trials, they can be easily overlooked as “mere anecdotes” [3]. However, case reports not only guide personalized treatment in clinical practice, but they also generate hypotheses for future clinical trials [4]. In recent years, integrating systematically collected data from the real world by using sophisticated clinical research methods has been expected to uncover hidden evidence [4]. Thus, patient case reports can be valuable sources of new information that may lead to vital research and advances in clinical practice, in turn improving patient outcomes [5]. Given that acupuncture involves complex and varied forms of treatment, it is necessary to carefully record what happens in clinical practice [3].

The “CAse REport (CARE) guidelines” were proposed to facilitate systematic reporting of information in case reports [1]. It is widely expected that implementation of the CARE guidelines will improve the completeness and transparency of case reporting [1]. Guidelines for reporting adverse events of acupuncture were proposed in 2004, but there was no specific guideline for case reports about acupuncture treatment [6]. Recently, the Korean version of the CARE guidelines checklist was implemented in case reporting of acupuncture treatment [7]. The STandards for Reporting Interventions in Clinical Trials of Acupuncture (STRICTA), originally developed to improve the completeness and transparency of the reporting of interventions in controlled trials of acupuncture, are now expected to expand to encompass a broad range of clinical evaluation designs, including case reports [8, 9]. Since STRICTA recommendations comprise a checklist that expanded the generic content of Item 4 (reporting of intervention) of the CONSORT statement for controlled trials, it would also be necessary to follow the STRICTA guidelines for reporting acupuncture interventions in case reports. Author guidelines in journals suggest that full details of the acupuncture treatment in case reports should follow the STRICTA criteria [3]. To the best of our knowledge, no study has investigated the reporting quality of case reports in acupuncture research in Korea based on the CARE and STRICTA guidelines.

Hence, we aimed to assess the current status of the reporting quality of case reports concerning acupuncture treatment in Korea based on the CARE and STRICTA guideline checklists.

## 2. Methods

### 2.1. Searching for and Selecting Case Reports

To assess the quality of reporting in acupuncture treatment case reports in Korea, all such reports were searched for in the Korean academic journal,* The Acupuncture (The Journal of Korean Acupuncture and Moxibustion Medicine Society)*. This journal is considered a core journal, which is highly cited in Korea [10]. Since this journal publishes acupuncture treatment case reports with separate subheadings, all case reports were retrieved based on the subheadings from January 2009 to September 2015. All case reports with acupuncture treatment as the intervention, regardless of the patient's diagnosis, were included in the analysis.

### 2.2. Data Extraction

Data were extracted independently by two assessors (Jeongjoo Kim and Yoon-Ji Eom) in accordance with prepared data extraction forms. As the STRICTA guidelines were originally developed to report the components of needling acupuncture, acupuncture was defined in this study as needle penetration of body points using manual and electrical stimulation [11]. To assess the quality of reporting of treatment components of acupuncture interventions in case reports, we used the revised version of the STRICTA guidelines published in 2010 [8]. Only the acupuncture-related information was extracted for analysis of the STRICTA items. The CARE guideline and STRICTA guidelines were converted into 31 and 15 checklist items, respectively, for data assessment. Before the evaluation, two assessors underwent training on each CARE and STRICTA item to ensure consistency in interpretation and scoring.

### 2.3. Evaluation of the CARE and SRTICTA Guideline Checklists

Items were worded closely to correspond to the original recommendations and rephrased as a series of questions. Each item from CARE and STRICTA was assessed as “yes” if it was included in the article or “no” if it was not. When at least one subitem was completely reported, the reporting item was counted as “yes.” The interrater reliability was calculated using Cohen's kappa statistic for all items combined (kappa = 0.834 for CARE items; kappa = 0.729 for STRICTA items), and disagreements were resolved by joint discussion with a third assessor (Younbyoung Chae).

The CARE and STRICTA index was calculated to summarize the overall completeness of reporting by summing the scores for the 31 items of the CARE checklist and 15 items of the STRICTA guidelines [11, 12].

## 3. Results

### 3.1. Included Case Reports

**Figure 1:**
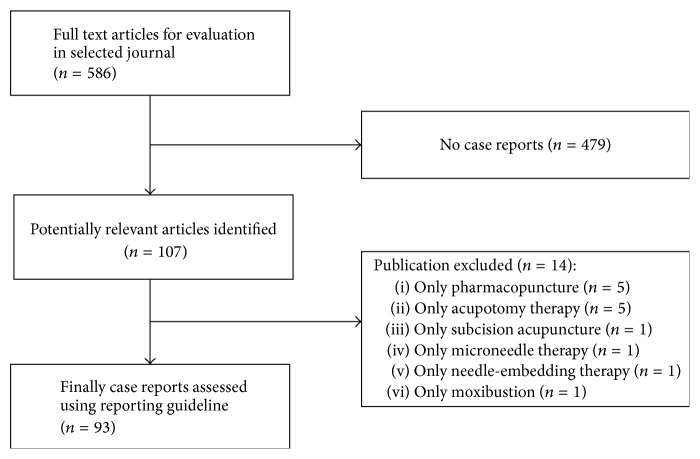
Flow chart of the articles identified, included, and excluded.

In total, 93 of 107 screened case reports of acupuncture treatment were included for the assessment of reporting quality. Studies that combined acupuncture with other interventions were included, but those assessing only other interventions were excluded (*n* = 14) (Figure 1).

### 3.2. Quality of Reporting with CARE Guideline Items

The overall quality of reporting was relatively high (mean = 75.4%, 95% CI: 74.4 to 76.4) (Table 1). The CARE index was 23.4 (95% CI: 23.1 to 23.7). Items with markedly incomplete reporting (less than 50%) were diagnostic challenges (number 16, 2.2%), diagnostic reasoning including consideration of other diagnoses (number 17, 12.9%), prognostic characteristics (number 18, 25.8%), changes in intervention with rationale provided (number 21, 25.8%), intervention adherence and tolerability (number 24, 0%), adverse and unanticipated events (number 25, 16.1%), patient perspective (number 30, 29.0%), and informed consent (number 31, 12.9%).

### 3.3. Quality of Reporting with STRICTA Guideline Items

The quality of reporting of acupuncture interventions in case reports was evaluated according to the STRICTA guidelines. The overall quality of reporting was acceptable (mean = 67.7%, 95% CI: 64.8 to 70.6) (Table 2). The STRICTA index was 10.2 (95% CI: 9.7 to 10.6). Items with markedly incomplete reporting (less than 50%) were acupuncture regimen variation (number 3, 33.3%), depth of insertion (number 6, 46.2%), response sought (number 7, 23.7%), setting and context (number 14, 18.3%), and description of acupuncturists (number 15, 23.7%).

## 4. Discussion

A total of 93 case reports of acupuncture treatment in Korea were appraised in detail based on CARE and STRICTA guidelines. This study systematically illustrates the current reporting quality of case reports of acupuncture treatment. Quality of reporting was generally acceptable, but some items require further improvement. Our findings reveal the current status of the quality of reporting in case reports of acupuncture treatment in Korea and provide information that may facilitate the transparency and completeness of the reporting of case reports. Information obtained from transparent and detailed case reports would help provide a stronger basis for elucidation of the scope and effectiveness of acupuncture treatment, which in turn would be helpful in expanding the field of acupuncture research, as well as in developing further guidelines regarding clinical acupuncture practice.

With the prominent increase of case reports in medical journals, CARE guidelines provide a framework for a systematic reporting standard [4]. In the current study, the overall completeness of reporting of case reports in Korea was relatively high (75.4%), but several items were still lacking in the majority of the acupuncture treatment case reports. The reporting of diagnostic assessment items, such as diagnostic challenges (number 16), diagnostic reasoning including consideration of other diagnoses (number 17), and prognostic characteristics (number 8), was remarkably incomplete. Low quality reporting, particularly in the diagnostic assessment items, might be due to the dual medical system in Korea, in which Korean medical doctors are very limited in terms of their ability to use medical examination equipment. Concerning therapeutic intervention, changes in intervention (number 21) were reported in about 25.8% of the included case reports. Among the follow-up and outcome criteria, none of the acupuncture treatment case reports reported intervention adherence, and tolerability and adverse and unanticipated events were reported in only 16.1% of the included case reports. Items related to patient perspective (number 30) and informed consent (number 31) were markedly underreported. Collectively, these items should be more carefully presented in case reports.

In the present study, the quality of the reporting of intervention details based on STRICTA items was generally acceptable (67.7%) but still less complete than that based on CARE items. Among the poorly reported items (under 50%), acupuncture regimen variation (number 3) was reported by about one-third of the case reports. Insertion depth (number 6) and* de qi* response (number 7) were reported relatively infrequently, in 46.2% and 23.7% of case reports, respectively. Insertion depth and* de qi* response are important as the main specific components of acupuncture treatment [13–15]. To explore the causal relationship between acupuncture and outcome, these components should be much improved in acupuncture treatment case reports. Setting and context (number 14) and description of the acupuncturists (number 15) were considerably underreported, at 18.3% and 23.7%, respectively. These two variables are known to be nonspecific components of acupuncture treatment, and these items are considered less important in clinical trials [16–18]. One potential way to minimize the possible involvement of nonspecific effects in acupuncture treatment is through complete reporting of these items. Based on the STRICTA index in Korea, the reporting quality of case reports in the current study was similar to that of clinical trials in other studies [10, 11]. However, only 4 out of 93 case reports adopted STRICTA guidelines and reported all items. We strongly suggest that intervention details of acupuncture treatment in case reports be described according to STRICTA criteria.

Reporting guidelines play an important role in improving the quality of papers in clinical trials [11, 19–21]. The CARE guidelines were developed and translated into Korean to improve the completeness and transparency of case reports; however, they are not limited specifically to case reports of acupuncture treatment [1]. Recently, case reports involving traditional Chinese medicine (CARC) were developed based on a review of the general reporting quality of those reports, and through internal discussion by experts [22]. The CARC recommendations covered all traditional Chinese medicine interventions including Chinese herbal interventions, acupuncture, and moxibustion. Moreover, these recommendations suggest that items should include diagnosis by traditional Chinese medicine-based methods, according to symptoms, signs, and the characteristics of the tongue and pulse. According to the survey, 67.4% of case reports included the traditional Chinese medicine terms for diseases, and 88.9% reported syndrome differentiation [23]. These efforts in the CARC recommendations might reflect the perspective that acupuncture treatment is determined not just by diagnosis based on Western medicine, but also by pattern identification based on traditional Chinese medicine.

The reporting quality of case reports based on CARE was relatively good in the Korean literature, but the pattern identification process for determining the acupuncture treatment procedure was still poorly reported. The lack of reports, however, does not reflect the current process of acupuncture treatment, in which pattern identification is not in any way undervalued. Pattern identification is accepted as one of the key components for deciding the acupuncture treatment approach. For instance, in Saam acupuncture in Korea, pattern identification enables the selection of acupoints that are not only proximal, but also distal, to the symptom-related organs or body parts [24, 25]. Considering that the process of determining the method of acupuncture treatment is based on pattern identification in clinical practice, it is necessary to include additional, crucial information about the characteristics of the tongue and pulse, as well as pattern identification based on symptoms and signs. New guidelines specifically tailored toward case reports of acupuncture treatment and reflecting the whole process of clinical practice will be required in the future.

This study had several limitations. First, the results might not fully represent all Korean journals, as the case reports that were included were extracted from a single journal. Because this journal alone has adopted reporting guidelines, the quality of reports of randomized controlled trials in traditional medicine journals in Korea was assessed in a separate, previous study [10]. Considering the representativeness of this journal, of the field of acupuncture research in Korea, it is reasonable to assess the quality of reporting of case reports according to the papers published therein. Second, we did not compare the compliance rate for the quality of reporting following the publication of the CARE and STRICTA recommendations in Korea over time, because all case reports in this study were published after 2009. However, our findings could provide valuable information about the current, overall reporting quality for acupuncture case reports in Korea. Furthermore, a future study will be needed to compare changes in the reporting quality of case reports after endorsement of the CARE and STRICTA guidelines.

Classical medical texts, such as* Shanghanlun* and* Linzhengzhinanyian*, are enclosed with several case reports in East Asian medicine [22]. They include a delicate reporting form to record the diagnosis, principles of treatment, therapeutic outcome, and prognosis of practical cases. Owing to low reporting quality, however, the ability to study and analyze the underlying principles of East Asian medicine based on these case reports remains limited. Case reports are inherently unable to exclude the possibility that outcomes are due to natural factors or the effects of another intervention [3]. Without a relevant control group, it is difficult to ascertain the extent to which a given outcome was due specifically to the effects of acupuncture treatment and how much was attributable to nonspecific effects. However, most patients have symptoms that do not accord exactly with the diagnostic criteria strictly defined by researchers [26]. From the perspective of patient-centered medicine, it is emphasized that the patient is more than the sum of his or her diseases. As Hippocrates stated “I would rather know the person who has the disease than the disease the person has” [27]. Case reports are most valuable in the context of patient-centered medicine, since they describe the personal experiences of a specific practitioner and disseminate valuable clinical information about patients in a more vivid manner.

In sum, the overall reporting quality of case reports was generally acceptable, but several crucial items remained substantially underreported in Korean acupuncture treatment case reports. Endorsement of the CARE and STRICTA guidelines is needed to improve the completeness of reporting of acupuncture treatment-based case reports. Case reports with more transparency in their content, as well as sufficiently detailed information, would be more useful not only for the care of individual patients, but also for healthcare providers and the broader medical community. Our findings will be helpful in developing more appropriate reporting guidelines for case reports of acupuncture treatment.

## Figures and Tables

**Table 1:** Percentage of case reports with complete reporting of CARE items.

		Item	*n*/*N*	(%)
Number 1	Title	The words “case report”	93/93	100.0
Number 2	Keywords	Key elements	93/93	100.0
Number 3	Abstract	Introduction: what does this add?	93/93	100.0
Number 4		Case presentation: main symptoms	47/93	50.5
Number 5		Case presentation: main clinical findings	91/93	97.8
Number 6		Case presentation: main diagnoses and interventions	93/93	100.0
Number 7		Case presentation: main outcomes	93/93	100.0
Number 8		Conclusion: main “take-away” lessons	93/93	100.0
Number 9	Introduction	Brief background summary of this case	93/93	100.0
Number 10	Patient information	Demographic information	93/93	100.0
Number 11		Main symptoms of the patient	93/93	100.0
Number 12		Medical, family, and psychosocial history	92/93	98.9
Number 13	Clinical findings	Physical examination findings	93/93	100.0
Number 14	Timeline	Depicts important dates and times	77/93	82.8
Number 15	Diagnostic assessment	Diagnostic methods	83/93	89.2
*Number 16*		*Diagnostic challenges*	*2/93*	2.2^*∗*^
*Number 17*		*Diagnostic reasoning including other diagnoses considered*	*12/93*	12.9^*∗*^
*Number 18*		*Prognostic characteristics*	*24/93*	25.8^*∗*^
Number 19	Therapeutic intervention	Type of intervention (e.g., pharmacologic, surgical, preventive)	93/93	100.0
Number 20		Administration of intervention (e.g., dosage, strength, duration)	93/93	100.0
*Number 21*		*Changes in intervention (with rationale)*	*24/93*	25.8^*∗*^
Number 22	Follow-up and outcomes	Clinician and patient-assessed outcomes	93/93	100.0
Number 23		Important follow-up test results	93/93	100.0
*Number 24*		*Intervention adherence and tolerability*	*0/93*	0^*∗*^
*Number 25*		*Adverse and unanticipated events*	*15/93*	16.1^*∗*^
Number 26	Discussion	Strengths and limitations of the management of this case	93/93	100.0
Number 27		The relevant medical literature	93/93	100.0
Number 28		The rationale for conclusions (assessments of cause and effect)	93/93	100.0
Number 29		The main “take-away” message	93/93	100.0
*Number 30*	*Patient perspective*	*Patient perspective or experience*	*27/93*	29.0^*∗*^
*Number 31*	*Informed consent*	*Informed consent*	*12/93*	12.9^*∗*^

		Average	70.3/93	75.4
		CARE index: mean (95% CI)	75.4 (74.4 to 76.4)	

Values are the number of case reports that included the item divided by the total number of eligible case reports; ^*∗*^less than 50%.

**Table 2:** Percentage of case reports with complete reporting of STRICTA items.

	Item	*n*/*N*	(%)
Number 1	Style of acupuncture	93/93	100.0
Number 2	Reasoning for treatments	69/93	74.2
*Number 3*	*Acupuncture regimen variation*	*31/93*	33.3^*∗*^
Number 4	Number of needles	76/93	81.7
Number 5	Name of points	90/93	96.8
*Number 6*	*Depth of insertion*	*43/93*	46.2^*∗*^
*Number 7*	*Response sought (e.g., de qi)*	*22/93*	23.7^*∗*^
Number 8	Needle stimulation (e.g., manual, electrical)	65/93	69.9
Number 9	Needle retention time	79/93	84.9
Number 10	Needle type (diameter, length, etc.)	88/93	94.6
Number 11	Number of treatment sessions	80/93	86.0
Number 12	Frequency and duration of treatment sessions	84/93	90.3
Number 13	Details of other interventions	85/93	91.4
*Number 14*	*Setting and context*	*17/93*	18.3^*∗*^
*Number 15*	*Description of acupuncturists*	*22/93*	23.7^*∗*^

	Average	62.9/93	67.7
	STRICTA index: mean (95% CI)	10.2 (9.7 to 10.6)	

Values are the number of case reports that included the item divided by the total number of eligible case reports; ^*∗*^less than 50%.

## References

[1] Gagnier J. J., Kienle G., Altman D. G. (2013). The CARE guidelines: consensus-based clinical case reporting guideline development. *BMJ Case Reports*.

[2] Hauben M., Aronson J. K. (2007). Gold standards in pharmacovigilance: the use of definitive anecdotal reports of adverse drug reactions as pure gold and high-grade ore. *Drug Safety*.

[3] White A. (2004). Writing case reports—author guidelines for acupuncture in medicine. *Acupuncture in Medicine*.

[4] Riley D. (2013). Case reports in the Era of clinical trials. *Global Advances in Health and Medicine*.

[5] Cohen H. (2006). How to write a patient case report. *American Journal of Health-System Pharmacy*.

[6] Peuker E., Filler T. (2004). Guidelines for case reports of adverse events related to acupuncture. *Acupuncture in Medicine*.

[7] Lee S. M., Shin Y. S., Nam D. W., Choi D. Y. (2015). Korean translation of the CARE guidelines. *The Acupuncture*.

[8] MacPherson H., Altman D. G., Hammerschlag R. (2010). Revised STandards for Reporting Interventions in Clinical Trials of Acupuncture (STRICTA): extending the CONSORT statement. *PLOS Medicine*.

[9] MacPherson H., White A., Cummings M., Jobst K., Rose K., Niemtzow R. (2001). Standards for reporting interventions in controlled trials of acupuncture: the STRICTA recommendations. *Complementary Therapies in Medicine*.

[10] Choi J., Jun J. H., Kang B. K., Kim K. H., Lee M. S. (2014). Endorsement for improving the quality of reports on randomized controlled trials of traditional medicine journals in Korea: a systematic review. *Trials*.

[11] Kim K. H., Kang J. W., Lee M. S., Lee J.-D. (2014). Assessment of the quality of reporting in randomised controlled trials of acupuncture in the Korean literature using the CONSORT statement and STRICTA guidelines. *BMJ Open*.

[12] Han C., Kwak K.-P., Marks D. M. (2009). The impact of the CONSORT statement on reporting of randomized clinical trials in psychiatry. *Contemporary Clinical Trials*.

[13] Chae Y., Olausson H. (2017). The role of touch in acupuncture treatment. *Acupuncture in Medicine*.

[14] Jung W. M., Shim W., Lee T. (2016). More than DeQi: spatial patterns of acupuncture-induced bodily sensations. *Frontiers in Neuroscience*.

[15] Langevin H. M., Wayne P. M., MacPherson H. (2011). Paradoxes in acupuncture research: strategies for moving forward. *Evidence-Based Complementary and Alternative Medicine*.

[16] Chung H., Lee H., Chang D.-S. (2012). Doctor’s attire influences perceived empathy in the patient-doctor relationship. *Patient Education and Counseling*.

[17] Kaptchuk T. J., Kelley J. M., Conboy L. A. (2008). Components of placebo effect: randomised controlled trial in patients with irritable bowel syndrome. *British Medical Journal*.

[18] Lee I.-S., Wallraven C., Kong J. (2015). When pain is not only pain: inserting needles into the body evokes distinct reward-related brain responses in the context of a treatment. *Physiology and Behavior*.

[19] Lu L.-M., He J., Zeng J.-C., Liao M.-X., Jia C., Pan H.-H. (2016). Impact evaluation of CONSORT and STRICTA guidelines on reporting quality for randomized controlled trials of acupuncture conducted in China. *Chinese Journal of Integrative Medicine*.

[20] Ma B., Chen Z., Xu J. (2016). Do the CONSORT and STRICTA checklists improve the reporting quality of acupuncture and moxibustion randomized controlled trials published in chinese journals? a systematic review and analysis of trends. *PLOS ONE*.

[21] Prady S. L., Richmond S. J., Morton V. M., MacPherson H. (2008). A systematic evaluation of the impact of STRICTA and CONSORT recommendations on quality of reporting for acupuncture trials. *PLoS ONE*.

[22] Fu S.-F., Cheng C.-W., Zhang L. (2016). Consensus-based recommendations for case report in Chinese medicine (CARC). *Chinese Journal of Integrative Medicine*.

[23] Fu S.-F., Kun W., Zeng X.-X. (2016). Urgent need to improve the quality of case report in traditional chinese medicine: assessment on reporting quality of 3,417 cases. *Chinese Journal of Integrative Medicine*.

[24] Lee T., Jung W., Lee I. (2014). Data mining of acupoint characteristics from the classical medical text: DongUiBoGam of korean medicine. *Evidence-Based Complementary and Alternative Medicine*.

[25] Park M., Kim S. (2015). A modern clinical approach of the traditional Korean Saam acupuncture. *Evidence-Based Complementary and Alternative Medicine*.

[26] Glasziou P. P., Irwig L. M. (1995). An evidence based approach to individualising treatment. *The BMJ*.

[27] Bensing J. (2000). Bridging the gap: the separate worlds of evidence-based medicine and patient-centered medicine. *Patient Education and Counseling*.

